# Supplementary material for: WordCommentsAnalyzer: A windows software tool for qualitative research
Source: F1000Res. 2018 Sep 4;7:536. Originally published 2018 May 3. [Version 2] doi: 10.12688/f1000research.14819.2 (PMC6137411; doi:10.12688/f1000research.14819.2)
Supplement: Supplementary file 3 [file f1000research-7-17614-s0002.tgz › b2a79496-4ada-47c4-9682-a99ce867836d.zip!/Eight Iranian Health Journals/Journal of Health System Research _42.docx]

© Journal of Health System Research . This work is licensed under CC BY-NC 4.0 (https://creativecommons.org/licenses/by-nc/4.0/).

Volume & Issue: دوره 7، شماره 3؛ 1390

Title: PRECEDE بررسي عوامل مرتبط با كيفيت زندگي زنان يائسه شهر اصفهان بر اساس مرحله تحليل رفتاري الگوي

http://hsr.mui.ac.ir/index.php/jhsr/article/view/245

مقدمه: در مطالعات گوناگون نشان داده شده است كه كيفيت زندگي زنان، به دنبال وقوع يائسگي دست‌خوش تغييرات اساسي مي‌گردد. عوامل متعددي از قبيل عوامل رواني- اجتماعي با كيفيت زندگي در دوران يائسگي مرتبط هستند. هدف اين مطالعه بررسي برخي عوامل مرتبط با كيفيت زندگي زنان يائسه اصفهاني بر اساس مرحله تحليل رفتاري الگوي پرسيد است. روش‌ها: اين مطالعه به صورت مقطعي و از طريق نمونه‌گيري تصادفي طبقه‌اي در ميان 200 زن يائسه سالم شهر اصفهان اجرا گرديد. اطلاعات مورد نياز توسط دو پرسش‌نامه معتبر و پايا جمع‌آوري گرديد (يك پرسش‌نامه كيفيت زندگي و پرسش‌نامه ديگر عوامل مربوط به سازه تحليل رفتاري الگوي پرسيد را مي‌سنجيد). تجزيه و تحليل داده‌ها از طريق نرم‌افزار 18 SPSSو با استفاده از آمار توصيفي و تحليلي صورت گرفت. يافته‌ها: ميانگين سني افراد مورد مطالعه 77/4 ± 74/55 سال و میانگین سن وقوع یائسگی 56/3 ± 20/50 سال به دست آمد. 5/30 درصد افراد مورد بررسي بي‌سواد بودند. 5 درصد از آن‌ها نيز تحصيلات دانشگاهي داشتند. در ضمن 81 درصد زنان متأهل و 90 درصد آن‌ها خانه‌دار بودند. آزمون همبستگي پيرسون، همبستگي مثبت و معني‌داري بين كيفيت زندگي با نوع نگرش نسبت به پديده يائسگي، خودكارآمدي درك‌شده، عوامل قادرساز و تقويت‌كننده نشان داد. همچنین كيفيت زندگي زنان يائسه با سن، سطح تحصيلات، وضعيت تأهل و وضعيت اشتغال آن‌ها ارتباط معني‌داري داشت. در ضمن اين آزمون، ارتباطي ميان كيفيت زندگي با آگاهي نسبت به يائسگي نشان نداد. نتيجه‌گيري: بر اساس نتايج اين مطالعه پيشنهاد مي‌شود كه فراهم‌كنندگان خدمات سلامتي، بايستي علاوه بر فراهم كردن آگاهي‌هاي لازم در مورد يائسگي، نسبت به خلق گروهاي حمايتي براي ايجاد نگرش مثبت و ارتقای رفتارهاي سالم‌تر در زنان يائسه همكاري نمايند.

Title: بررسی ارتباط برخی عوامل روانی- اجتماعی با رفتارهای تبعیت از درمان بیماران همودیالیز شهر اصفهان: چارچوبی مفهومی بر اساس تئوری شناختی- اجتماعی

http://hsr.mui.ac.ir/index.php/jhsr/article/view/244

مقدمه: حفظ سلامت بیماران تحت همودیالیز بستگی به تبعیت از توصیه‌های درمانی دارد. عدم تبعیت درمانی این بیماران، به صورت مستقیم با پیامدهای بالینی وخیم‌تر مرتبط می‌باشد. هدف از پژوهش حاضر، شناسایی روابط بین افسردگی، حمایت اجتماعی درک‌ شده، خودکارامدی و تعامل بیمار با پرسنل درمانی با رفتارهای تبعیت از درمان در بیماران همودیالیز بر اساس تئوری شناختی- اجتماعی بود. روش‌ها: مطالعه حاضر مقطعی است و بر روي 160 بیمار مراکز همودیالیز شهر اصفهان انجام شد. شیوه نمونه‌گیری، تصادفی منظم و متناسب با حجم بود. ابزار جمع‌آوری اطلاعات شامل پرسش‌نامه افسردگی Beck، مقياس چند بعدي حمايت اجتماعي ادراك‌شده، راهبردهای ارتقای سلامتی، ابزار سنجش تعامل بیمار- پرسنل درمان و پرسش‌نامه خود گزارش‌دهی رفتارهای تبعیت درمانی بود. اطلاعات گردآوري شده توسط آزمون‌هاي توصیفی و همبستگي مورد تجزيه و تحليل قرار گرفت. یافته‌ها: 6/60 درصد از افراد مورد مطالعه مرد بودند. میانگین سنی افراد مورد مطالعه 04/15 ± 95/51 بود. میانگین نمره کل رفتارهای تبعیت از درمان 64/201 ± 48/861 در دامنه (1200-0) به دست آمد. بین عوامل روانی– اجتماعی مورد بررسی با رفتارهای کل تبعیت از درمان ارتباطی مشاهده نشد اما بین این عوامل با زیرمقیاس‌های رفتاری، ارتباط معنی‌دار مشاهده گردید (05/0 > P). همه این عوامل با نمره درک از اهمیت رفتار ارتباط معنی‌دار داشتند (001/0 > P). بین متغیرهای جمعیت‌شناختی همچون سن، جنس و وضعیت تأهل با مقیاس و زیرمقیاس رفتار تبعیت درمان ارتباط معنی‌دار مشاهده گردید (05/0 > P). نتیجه‌گیری: بر اساس نتایج به دست آمده به نظر می‌رسد متغیرهای روانی– اجتماعی مورد مطالعه از طریق نقش واسطه‌ای عامل درک از اهمیت رفتار بر رفتار پیامد مؤثرند که لزوم توجه بیشتر به این عامل در مطالعات آتی و طراحی مداخلات مبتنی بر تئوری احساس می‌گردد.

Title: ارتباط بازی‌های رایانه‌ای بر کیفیت زندگی در نوجوانان 15-12 ساله شهرستان برخوار

http://hsr.mui.ac.ir/index.php/jhsr/article/view/243

مقدمه: رشد سريع بازي‌هاي رايانه‌اي به شکل‌های گوناگون باعث اشغال بخش عمده‌ای از اوقات فراغت گروه‌هاي سني مختلف به ويژه كودكان و نوجوانان شده است. این امر می‌تواند اثرات مستقيم و غير مستقيمی بر ابعاد مختلف کیفیت زندگی از جمله وضعیت رواني، جسماني و روابط اجتماعی آن‌ها داشته باشد. بنابراین اين تحقیق با هدف بررسی ارتباط بازی‌های رایانه‌ای با کیفیت زندگی نوجوانان 15-12 ساله شهرستان برخوار انجام گرديد. روش‌ها: این تحقیق از نوع مقطعي و نمونه تحقیق شامل 444 دانش‌آموز دختر و پسر بود. روش نمونه‌گیری طبقه‌اي- دو مرحله‌اي، خوشه‌ای و متناسب با حجم نمونه بود که نمونه‌ها به طور تصادفی از بین دانش‌آموزان مقطع راهنمایی و اول دبیرستان شهرستان برخوار انتخاب شدند. ابزار گردآوري اطلاعات شامل دو ابزار الف) فرم کوتاه پرسش‌نامه كيفيت زندگي سازمان جهانی بهداشت (World health organization quality of life–BREF یا WHOQOL-BREF) و ب) پرسش‌نامه اطلاعات فردی مشتمل بر سؤالاتی در مورد نحوه گذران اوقات فراغت، تعداد ساعات، مکان و زمان انجام بازی رایانه‌ای بود. براي به دست آوردن نمايه توده بدني (Body mass index یا BMI) نیز قد و وزن دانش‌آموزان گروه نمونه اندازه‌گيري شد. جهت تجزیه و تحلیل اطلاعات از آزمون‌های همبستگی Pearson، همبستگی Spearman، 2χ ، تحلیل کوواریانس و t-test مستقل استفاده شد. یافته‌ها: میانگین کلی کیفیت زندگی در دانش‌آموزان 34/13 ± 11/67 بود. بين سن شروع بازي و نمره كلي كيفيت زندگي و 4 حيطه آن رابطه معنی‌دار وجود داشت (دامنه 18/0- تا 13/0- = r). بين درامد و وضعيت امكانات با نمره كلي كيفيت زندگي و 4 حيطه آن رابطه معنی‌دار مستقيم وجود داشت (001/0 P <). میانگین نمره کلی کیفیت زندگی، در کاربران بازی‌های رایانه‌ای 03/13 ± 27/68 و در گروهی که بازی‌های رایانه‌ای انجام نمی‌دادند، 69/13 ± 81/64 به دست آمد که این تفاوت معنی‌دار بود (01/0 = P). در حیطه‌های کیفیت زندگی بین دو گروه در ابعاد سلامت روان و سلامت محیط، نیز تفاوت معنی‌داری به دست آمد (05/0 > P). بين BMI و نوع بازي رایانه‌ای و همچنين بين BMI و تعداد ساعات بازي رايانه‌اي در روز رابطه معنی‌داري به دست نيامد. نتیجه‌گیری: انجام بازی‌های رایانه‌ای در مدت زمان کم و کنترل‌شده تحت نظارت والدین می‌تواند اثراتی مثبتی بر کیفیت زندگی کاربران نوجوان داشته باشد. در صورت پرداختن به آن در ساعات بیشتری از روز، در دراز مدت می‌تواند تأثیر معکوس داشته باشد.

Title: تحلیل پیامد حوادث عمده و تعیین سطح یکپارچگی ایمنی فرایند در واحد آب ترش پالایشگاه گازی با استفاده از روش آنالیز لایه‌های حفاظتی

http://hsr.mui.ac.ir/index.php/jhsr/article/view/242

مقدمه: توسعه منابع نفت و گاز، مخاطرات جدی فرایندی را به دنبال داشته و این صنایع را در زمره صنایع خطرناک قرار داده است. از این رو، امروزه رویکرد مبتنی بر پیش‌گیری، از طریق شناسایی و ارزیابی خطرها به روش‌های نوینی نظیر روش آنالیز لایه‌های حفاظتی (LOPA یا Layers of protection analysis) مورد استقبال قرار گرفته است. این مطالعه به بررسی و آنالیز پیامد حوادث عمده در واحد احيای آب ترش پالایشگاه‌های گازی پرداخته و کفایت تأثیر لایه‌های حفاظتی موجود در سیستم را بررسی کرده و در مواردی که تمهیدات ایمنی پیش‌بینی شده کافی نبوده است، پیشنهاداتی را در راستای ارتقای سطح ایمنی با استفاده از لایه‌های حفاظتی مستقل (فعال و واکنشی) و غیر مستقل ارایه کرده است. روش‌ها: این تحقیق مطالعه‌ای توصیفی بود که با استفاده از روش LOPA، پیامدهای ناشی از خطرهای غیر قابل قبول بررسی شد تا وضعیت خطر، پس از اعمال لایه‌های کنترلی و حفاظتی مشخص شود و در نهایت برای نقاط با خطر بالا اقدامات اصلاحی در قالب لایه‌های حفاظتی پیشنهاد و به دنبال آن کد یک‌پارچگی ایمنی در موارد لازم تعیین شد. یافته‌ها: بر اساس این مطالعه نقش لایه‌های حفاظتی موجود در کنترل خطرهای احتمالی تعیین و پیشنهادات لازم در قالب لایه‌های حفاظتی بیشتر تا کاهش میزان خطر به حد قابل قبول ارایه شد. همچنین تعیین سطح ایمنی یک‌پارچه مورد نیاز برخی تجهیزات نیز در موارد لازم انجام گردید. نتیجه‌گیری: در برخی موارد اثربخشی لایه‌های حفاظتی پیش‌بینی شده کافی نیست و ضروری است تا از لایه‌های حفاظتی بیشتری برای ارتقای پتانسیل پیش‌گیری سیستم‌های ایمنی فرایند استفاده شود.

Title: عوامل مرتبط با اتخاذ رفتار سالم بر اساس الگوی اعتقاد بهداشتی در بیماران سلی استان اصفهان

http://hsr.mui.ac.ir/index.php/jhsr/article/view/240

مقدمه: سل یک بیماری عفونی است که در اثر مجموعه مایکوباکتریوم‌های سلی ایجاد می‌شود. این بیماری یکی از شایع‌ترین بیماری‌های عفونی بوده، که شیوع آن تا حدی زیادی مرتبط با سبک زندگی افراد بیمار می‌باشد. هدف از مطالعه حاضر بررسی عوامل مرتبط با اتخاذ رفتار سالم بر اساس الگوی اعتقاد بهداشتی در بیماران سلی بوده است. روش‌ها: مطالعه حاضر یک مطالعه مقطعی بود که بر روی 196 نفر از بیماران سلی انجام شد. ابزار جمع‌آوری اطلاعات، پرسش‌نامه محقق ساخته‌ای با 47 گویه بود. Cronbach’s alpha پرسش‌نامه 9/73 تعیین شد. برای تعیین ارتباط بین سازه‌های مدل و اتخاذ رفتار سالم از آزمون همبستگی Pearson استفاده شد. یافته‌ها: میانگین نمره اتخاذ رفتار سالم در بیماران سلی (8/13 ± 52/87) بود. آزمون همبستگی Pearson بین سازه‌های آگاهی (536/0 = r)، حساسیت درک شده (36/0 = r)، منافع درک شده (347/0 = r) و موانع درک شده (143/0- = r) و نمره اتخاذ رفتار سالم در سطح کمتر از پنج صدم ارتباط معنی‌داری نشان داد. نتیجه‌گیری: رابطه مستقیمی بین سازه‌های آگاهی، حساسیت درک شده و منافع درک شده با اتخاذ رفتار سالم وجود داشت. نتایج مطالعه حاضر می‌تواند مبنایی برای استفاده در مداخلات آموزشی باشد. نتایج فوق مربوط به نمونه مطالعه حاضر است و تعمیم آن به گروه‌های دیگر با احتیاط صورت گیرد.

Title: عوامل مرتبط با انجام آزمایش خون مخفی در مدفوع جهت غربالگری سرطان کولورکتال، بر اساس سازه‌های مدل اعتقاد بهداشتی در افراد در معرض خطر متوسط در اصفهان

http://hsr.mui.ac.ir/index.php/jhsr/article/view/239

مقدمه: همه ساله نزدیک به یک میلیون مورد جدید، سرطان کولورکتال در سراسر جهان شناسایی می‌شود و نزدیک به نیمی از آن‌ها در اثر این بیماری جان خود را از دست می‌دهند. در ایران نیز سرطان‌های گوارشی شایع‌ترین نوع سرطان در میان مردان و دومین سرطان شایع، پس از سرطان پستان در زنان ایرانی محسوب می‌شوند. با پیش‌گیری ثانویه این بیماری می‌توان اقدامات لازم جهت درمان سریع و جلوگیری از گسترش آن به عمل آورد. آزمایش خون مخفی در مدفوع (Fecal occult blood test یا FOBT) به دلیل سهولت انجام و همچنین به لحاظ هزینه پایین آن، بر دیگر روش‌های غربالگری این بیماری اولویت دارد. بر این اساس انجام هر ساله آن برای همه افراد بالای 50 سال توصیه می‌شود. متأسفانه در کشور ما مشارکت افراد در برنامه‌های غربالگری سرطان کولورکتال پایین می‌باشد. از آن جایی که مدل اعتقاد بهداشتی پیش‌بینی کننده خوبی برای مؤلفه‌های مؤثر بر انجام این آزمایش می‌باشد، از این رو پژوهش حاضر با هدف تعیین عوامل مرتب با انجام آزمایش خون مخفی در مدفوع بر اساس سازه‌های مدل اعتقاد بهداشتی در افراد در معرض خطر متوسط صورت گرفت. روش‌ها: این مطالعه از نوع توصیفی- تحلیلی بود که بر روی 196 نفر بالای 50 سال در شهر اصفهان انجام شد. این افراد در دو گروه مراجعه‌ کننده به آزمایشگاه جهت انجام آزمایش خون مخفی در مدفوع و گروه غیر مراجعه‌ کننده به آزمایشگاه، به صورت مقایسه‌ای مورد پژوهش قرار گرفتند. جهت گردآوری اطلاعات از پرسش‌نامه خود ساخته و استاندارد شده مبتنی بر سازه‌های مدل اعتقاد بهداشتی استفاده شد. داده‌ها و اطلاعات به روش پرسشگری در آزمایشگاه برای گروه اول و درب منازل برای گروه دوم، جمع‌آوری شدند. اطلاعات جمع‌آوری شده از طریق نرم‌افزار SPSS و با به کارگیری آزمون‌های آماری 2χ، آنالیز واریانس دو طرفه، t-test، Mann-Whitney و Kruskal-Wallis مورد تجزیه و تحلیل قرار گرفت. یافته‌ها: مهم‌ترین راهنماهای عمل ذکر شده در گروه مراجعه‌ کننده به آزمایشگاه به ترتیب توصیه پزشک 54 درصد و انجام چکاپ 29 درصد بودند. بین کسانی که سابقه انجام آزمایش خون مخفی در مدفوع در سال گذشته را داشتند و افرادی که در گروه غیر مراجعه‌ کننده به آزمایشگاه این مراجعه را نداشتند، از لحاظ نمرات کسب شده از سازه‌های مدل اعتقاد بهداشتی (آگاهی، حساسیت درک‌ شده، شدت درک شده، موانع درک‌ شده، خودکارامدی درک شده و منافع درک شده) تفاوت معنی‌دار آماری وجود داشت. در گروه مراجعه کننده به آزمایشگاه، کسانی که سابقه انجام این آزمایش در سال گذشته را داشتند، از نظر نمرات کسب شده از سازه‌های شدت درک‌ شده، موانع درک‌ شده، خودکارامدی درک‌ شده و منافع درک شده با افرادی از همین گروه که سابقه انجام آزمایش را نداشتند، تفاوت معنی‌دار آماری وجود داشت. همچنین گروه مراجعه‌ کننده به آزمایشگاه از لحاظ نمرات کسب شده از سازه‌های مختلف مدل اعتقاد بهداشتی، به جز سازه موانع درک شده نمرات بالاتری را نسبت به گروه دیگر کسب کردند. نتیجه‌گیری: نتایج این پژوهش نشان داد که سازه‌های مدل اعتقاد بهداشتی، پیش‌بینی کننده خوبی برای عمل افراد در معرض خطر نسبت به انجام آزمایش خون مخفی در مدفوع می‌باشد. این آزمایش به عنوان یک روش غربالگری برای سرطان کولورکتال می‌باشد. این مهم لزوم اجرای برنامه‌های آموزشی وسیع و جامع با تمرکز بر سازه‌های مدل اعتقاد بهداشتی در این گروه از افراد را نمایان می‌کند. از آن جایی که آزمایش خون مخفی در مدفوع به عنوان ساده‌ترین، ارزان‌ترین و اولین راه تشخیص زود هنگام سرطان کولورکتال می‌باشد، دوره‌های آموزشی و بازآموزی برای پزشکان و سایر پرسنل بهداشتی جامعه در جهت بالا بردن میزان مشارکت افراد در انجام این آزمایش ضروری می‌باشد.

Title: تجزيه بيولوژيکي پرکلرواتيلن (PCE) در پساب صنعتي سنتتیک با استفاده از راکتور بي‌هوازی با بستر سيال (AMBR)

http://hsr.mui.ac.ir/index.php/jhsr/article/view/232

مقدمه: پرکلرواتيلن (Perchloroethylene یا PCE) از دسته هیدروکربن‌های آلیفاتیک کلردار است که کاربرد گسترده‌ای در بسیاری از صنایع دارد. پساب‌های صنعتی حاوی PCE به دلیل خطرات احتمالی ناشی از سرطان‌زایی این ترکیب باید تصفیه شوند. هدف از این مطالعه، تعیین قابلیت تجزيه بيولوژيکي پرکلرواتيلن در غلظت‌های بالاتر از مقادیر به کار رفته در سایر مطالعات مشابه، توسط یک راکتور بي‌هوازی با بستر سيال (AMBR) بود، که تاکنون برای تجزيه بيولوژيکي PCE مورد استفاده قرار نگرفته است. روش‌ها: در این مطالعه یک راکتور بي‌هوازی با بستر سيال (AMBR یا Anaerobic mobile batch reactor) در مقیاس آزمایشگاهی به حجم کل 10 لیتر با 4 محفظه برای تجزيه بيولوژيکي پرکلرواتيلن از سوبستره سنتتیک مورد استفاده قرار گرفت. راه‌اندازی راکتور به کمک لجن هاضم بی‌هوازی انجام شد. پس از راه‌اندازی، کارایی راکتور طی 4 مرحله از بارگذاری PCE معادل mgPCE/Ld 75/3 تا 75 مورد بررسی قرار گرفت. زمان ماند هیدرولیکی (HRT یا Hydraulic retention time) برابر 32 ساعت ثابت نگه داشته شد. یافته‌ها: بهترین بازده حذف COD به میزان 98 درصد در بارگذاری آلی COD معادل gCOD/Ld 1/3 به دست آمد. برای حذف PCE نیز بالاترین بازده به میزان 8/99 درصد در بارگذاری PCE معادل mgPCE/Ld 5/37 حاصل شد. میانگین بازده حذف COD و PCE در کل دوره فعالیت راکتور به ترتیب برابر 4/91 و 5/99 درصد بود. نتیجه‌گیری: راکتور بی‌هوازی با بستر سیال (AMBR)، در صورت انجام مطالعات تکمیلی در مقیاس کامل و استفاده از پساب صنعتی واقعی آلوده به پرکلرواتيلن یک روش ساده، کارامد و قابل اطمینان برای تصفیه پساب‌های صنعتی آلوده به این ترکیب است.

Title: غذایی DASH (کنترل کننده پرفشاری خون) و عوامل خطر بیماری‌های قلبی عروقی در بیماران مبتلا به دیابت نوع 2

http://hsr.mui.ac.ir/index.php/jhsr/article/view/231

مقدمه: تاكنون هيچ گونه شواهدي مبني بر اثر رژيم غذايي  (Dietary approaches to stop hyper) DASHبر روي فاكتورهاي خطر بيماري قلبي- عروقي در ميان بيماران ديابتي نوع 2 وجود نداشته است. از این رو، هدف از بررسی حاضر تعیین اثرات رژیم غذایی DASH بر روی عوامل خطر بیماری‌های قلبی- عروقی در میان بیماران مبتلا به دیابت نوع 2 بود. روش‌ها: یک کارآزمایی بالینی متقاطع و تصادفی بر روی 31 بیمار مبتلا به دیابت نوع 2 انجام شد. این مطالعه به مدت 8 هفته انجام شد و شرکت‌کنندگان به طور تصادفی برای استفاده از رژیم شاهد یا DASH تقسیم‌بندی شدند. یافته‌ها: پس از پیروی از الگوی غذایی DASH وزن بدن (007/0 = P) و دور کمر (002/0 = P) کاهش یافته بود. سطح گلوکز خون ناشتا (FPG) و HbA1C در رژیم DASH کاهش یافته بود (به ترتیب 04/0 = P و (mg/dl 3/6 ± 4/29- و 04/0 = P و درصد 1/0 ± 7/1-). بعد از رژیم DASH، میانگین تغییرات سطح HDL کلسترول افزایش (01/0 = P و mg/dl 9/0 ± 3/4) و سطح LDL کلسترول کاهش یافته بود (02/0 = P و mg/dl 5/3 ± 2/17-). علاوه بر آن، اثرات مفید DASH روی فشار سیستولیک و دیاستولیک بررسی شد (سیستولیک: 02/0 = P و mmHg 7/2 ± 1/3- در مقابل 5/3 ± 6/13-، دیاستولیک: 04/0 = P و mmHg 3/3 ± 7/0- در مقابل 6/2 ± 5/9-). نتیجه‌گیری: در میان بیماران مبتلا به دیابت، رژیم DASH اثرات مفیدی را بر خطر بیمارهای قلبی- عروقی داشت.

Title: تأثیر روابط خانوادگی بر بهداشت روانی فرزندان در شهر اصفهان

http://hsr.mui.ac.ir/index.php/jhsr/article/view/225

مقدمه: خانواده با فراهم نمودن روابط نزدیک و مثبت می‌تواند نقش مؤثری بر ارتقای بهداشت روانی افراد داشته باشد. پژوهش حاضر با هدف بررسی تأثیر روابط خانوادگی بر سلامت روانی جوانان اصفهان در سال 1388 صورت گرفت. روش‌ها: روش پژوهش پیمایش و ابزار جمع‌آوری اطلاعات پرسش‌نامه 80 سؤالی بود. جامعه آماری تحقیق شامل همه جوانان 19-15 ساله شهر اصفهان بود که از بین آن‌ها 384 نفر با شیوه نمونه‌گیری سهمیه‌ای به عنوان نمونه انتخاب شدند. برای سنجش سلامت روانی از پرسش‌نامه 28 سؤالی سازمان بهداشت جهانی (88/0 = ضریب پایایی) و برای سنجش روابط خانوادگی از شش شاخص مشارکت، اعتماد، انسجام، حمایت اقتصادی، حمایت عاطفی و کنترل و نظارت خانواده (90/0 = ضریب پایایی) استفاده شد. برای تجزیه و تحلیل اطلاعات مربوط به متغیرهای وابسته و مستقل، از روش‌های آماری ضریب همبستگی، ضریب تعیین، آزمون تحلیل واریانس و برای بررسی روابط علی بین متغیرها از معادله ساختاری استفاده شد. یافته‌ها: روابط خانوادگی سازنده، تأثیر مثبت و معنی‌داری بر سلامت روانی افراد داشتند (63/0 = α). تأثیر مؤلفه‌های روابط خانوادگی نیز همگی مثبت و معنی‌دار بودند (اعتماد 255/0 = 2R، حمایت اقتصادی 201/0 = 2R، حمایت عاطفی 184/0 = 2R، انسجام 192/0 = 2R، مشارکت 116/0 = 2R، کنترل و نظارت خانواده 116/0 = 2R). همچنین پایگاه اقتصادی- اجتماعی خانواده بر سلامت روانی جوانان تأثیر مثبت و معنی‌دار داشت، ولی بین جنسیت و سلامت روانی رابطه معنی‌دار وجود نداشت. نتیجه‌گیری: این مطالعه نشان داد که متغییرهای اساسی در خانواده‌‌های همگن و همسو تأثیر مثبت شایانی بر سلامت بچه‌‌ها دارد. بنابراین لازم است که خانواده‌ها تلاش کنند چنین ارتباطات مثبت در خانواده ایجاد کنند. همان طور که در سایر گروه‌ها نیز نشان داده شد، ارتباطات مثبتی بین سلامت روان و روش کار همگن در مدارس برای دانش‌آموزان وجود دارد.

Title: بررسي عوامل مؤثر بر انتخاب روش زايمان در زنان باردار شكم اول مراجعه كننده به مراكز بهداشتي درماني اردبيل

http://hsr.mui.ac.ir/index.php/jhsr/article/view/208

مقدمه: مکانیسم انجام زایمان یک فرایند خودبه‌خودی و بدون نیاز به مداخله می‌باشد به جز در مواردی که جان مادر یا نوزاد در خطر باشد، که در این صورت با انجام عمل جراحی سزارین به نجات جان مادر یا نوزاد کمک می‌شود. متأسفانه امروزه شیوع سزارین در اکثر کشورهای پیشرفته، به بیش از 50 درصد رسیده است، در حالی که سازمان بهداشت جهانی تأکید دارد سزارین تنها بر اساس اندیکاسیون انجام شود. در اين پژوهش، علل و عوامل مؤثر بر قصد انتخاب روش زايمان در زنان باردار شکم اول مورد بررسي قرار گرفت. روش‌ها: اين مطالعه توصیفی- تحلیلی، آینده‌نگر بود. جامعه آماری مطالعه شامل زنان باردار شکم اول مراجعه کننده به مراکز بهداشتی درمانی اردبیل بود که تعداد 396 نفر از زنان در سه ماهه آخر بارداري با نمونه‌گیری دو مرحله‌ای انتخاب شدند. براي جمع‌آوري اطلاعات، پرسش‌نامه‌اي مشتمل بر 4 سؤال است كه در 6 قسمت طراحي شده بود. روایی پرسش‌نامه توسط استادان متخصص و پایایی آن توسط آزمون Cronbach's alpha مورد تأييد قرار گرفت. به منظور تجزيه و تحليل داده‌ها از نرم‌افزار آماري 15SPSS و آزمون‌های Chi-square و Paired t استفاده شد. یافته‌ها: ميانگين سن مادران مراجعه كننده به مراكز بهداشتي اردبيل 2/3 ± 8 /21 سال بود و اكثر مادران (6/34 درصد) تحصيلات ديپلم داشتند. 7/70 درصد مادران ابراز كردند كه به احتمال زیاد یا به طور قطعی با روش زايمان طبيعي، زايمان می‌كنند؛ اما در ختم بارداري، روش سزارين با 6/58 درصد بيشترين روش انتخاب شده و توصيه پزشك با 6/36 درصد بيشترين عامل تأثیرگذار روی انتخاب روش زايمان بود. نگرش زنان نسبت به سزارین در قبل و بعد از زایمان کاهش معنی‌داری داشت (05/0 > P). نتیجه‌گیری: عوامل اجتماعی و انگيزه براي اطاعت از نرم‌های انتزاعی به ویژه از پزشک و پرسنل بهداشتی درمانی، بر روی قصد نهایی برای انجام رفتارهای مرتبط با سلامت، تأثیر بسزایی دارند. با توجه به این فاكتورهاي اجتماعي، مي‌توان با ارتقای آگاهي و نگرش مادران نسبت به زايمان طبيعي، در تقويت قصد مادران در انجام زايمان و حمايت‌هاي محيطي مادر در انتخاب روش زايمان طبيعي، مؤثرتر واقع شد.

Title: مطالعه تشخیصی گونه‌های قارچی درماتوفیتی در محوطه استخرهای شنای سرپوشیده شهر یزد

http://hsr.mui.ac.ir/index.php/jhsr/article/view/246

مقدمه: استفاده از استخرهای شنا یکی از راه‌های انتقال عوامل عفونت‌های قارچی سطحی و جلدی است. این تحقیق با هدف تعیین میزان آلودگی قارچ‌های درماتوفیت، pH و ارزیابی میزان کلر باقی‌مانده در استخرهای سرپوشیده شهر یزد در سال 1388 انجام گرفت. روش‌ها: در این مطالعه توصیفی- مقطعی چهار استخر شنای سرپوشیده شهر یزد به صورت تصادفی انتخاب شدند. سپس تعداد 100 نمونه از نواحی مختلف استخر شنا با استفاده از روش موکت استریل نمونه‌گیری شدند. جهت جداسازی و تشخیص آلودگی قارچی درماتوفیتی از کشت بر روی محیط میکوزل آگار و تشخیص گونه‌های قارچی استفاده شد. همچنین میزان pH و کلر آزاد باقی‌مانده آب استخرها نیز اندازه‌گیری شد. یافته‌ها: از مجموع 100 نمونه، 7 نمونه دارای کشت مثبت قارچ‌های بیماری‌زا درماتوفیتی بودند. این نمونه‌ها در نواحی مختلف استخرهای شنا از جمله رختكن، سونا و پاشویه وجود داشتند. قارچ‌های بیماری‌زا انسان‌دوست تريكوفيتون منتاگروفيت (6 مورد) و اپیدرموفیتون فلوکوزوم (1 مورد) از درماتوفیت‌های جدا شده و بیشترین موارد کشت مثبت مربوط به فصل تابستان بودند. میزان pH بین 8/7 تا 2/8 و میانگین کلر آزاد باقی‌مانده آب نیز از 4/0 تا 7/0 میلی‌گرم بر لیتر متغیر بود. نتیجه‌گیری: از آن جایی که جدا شدن آلودگی‌های قارچی درماتوفیتی انسان‌دوست از استخرهای مورد بررسی نشان‌دهنده انتشار عوامل قارچی بیماری‌زا از شناگران به محیط استخر می‌باشد؛ از این رو رعایت بهداشت فردی توسط شناگران، جلوگیری از ورود افراد دارای علایم بیماری‌های قارچی به استخر و انجام ضدعفونی مناسب‌تر محوطه در این استخرها ضرورت دارد.

Title: بررسي تغييرات كورتيزول ادراري كارگران در معرض صدا در كارخانه نساجي سپاهان لايه اصفهان

http://hsr.mui.ac.ir/index.php/jhsr/article/view/198

مقدمه: صدا يكي از مهم‌ترين عوامل زيان‌آور در محيط كار است و نقش بسزايي در به هم زدن تعادل فيزيولوژيكي بدن دارد. پژوهش‌های انجام يافته نشان مي‌دهد كه از نظر كارگران بزرگ‌ترين مشكل موجود در محيط كار آلودگي صوتي مي‌باشد. سر و صدا علاوه بر آثار سوء بر روي سيستم شنوايي، به عنوان يك عامل استرس‌زا ممكن است سبب افزايش فشار خون، بروز مشكلات قلبي- عروقي، تحريك اعصاب، اضطراب و اختلال در ترشح هورمون‌هاي بدن به ويژه كورتيزول شود. هدف از اين مطالعه، بررسي اثرات صدا بر روي ميزان ترشح كورتيزول ادرار كارگران بود. روش‌ها: این مطالعه از نوع تجربي و تحليلي و آينده‌نگر بود. جمعيت مورد مطالعه را كارگران كارخانه سپاهان لايه اصفهان تشكيل دادند. از بيست نفر كارگر مرد سالم كه افت شنوايي نداشتند و همه به طور مداوم در شيفت صبح كار مي‌كردند، سه بار در روز (در ساعات 9 صبح، 30/11 صبح و 3 بعد از ظهر) و در دو روز مطالعه، نمونه ادرار گرفته شد. طي روز اول كارگران از هيچ گونه وسيله حفاظت شنوايي استفاده نمي‌كردند، اما در روز دوم از كارگران خواسته شد كه از ايرماف استفاده كنند. ميزان كورتيزول ادرار با استفاده از دستگاه RIA  (Radioimmunoassay)و كراتينين ادرار نيز با روش JAFFE با استفاده از دستگاه اتوآنالايزر اندازه‌گيري شد. یافته‌ها: ميانگين كورتيزول ادرار كارگران در روز اول مطالعه و بدون استفاده از وسيله حفاظت شنوايي g/mgCratinineµ 105/0 و در روز دوم مطالعه و با استفاده از ايرماف g/mgCratinineµ 076/0 بود. ميانگين كورتيزول ادرار كارگران بر حسب g/mgCratinineµ در ساعات 9 صبح، 30/11 صبح و 3 بعد از ظهر در روز اول مطالعه به ترتيب 142/0، 119/0 و 052/0 و در روز دوم به ترتيب 103/0، 086/0 و 041/0 اندازه‌گيري شد. نتیجه‌گیری: نتايج آزمون  Paired tنشان داد كه ميانگين ميزان كورتيزول ادرار كارگران در دو روز مطالعه و همچنین در ساعات مختلف در دو روز مورد مطالعه اختلاف معني‌داري دارد (05/0 > P). در اين پژوهش به غير از عامل كاهش صدا در دو روز مطالعه، هيچ تغيير ديگري در محيط كار كارگران داده نشد. نتايج نشان داد كه به دنبال كاهش صدا در حدود 3/29 دسي‌بل (به دليل استفاده از ايرماف) كاهش قابل ملاحظه‌اي در ميزان كورتيزول و در نتيجه واكنش‌هاي استرسي رخ داده است و در نتيجه مقدار كورتيزول در روز اول (بدون استفاده از ايرماف) نسبت به روز دوم (با استفاده از ايرماف) به صورت معني‌داري كاهش پيدا كرد (05/0 > P). بنابراين نتايج اين مطالعه فوايد استفاده از وسايل حفاظت شنوايي مانند ايرماف در كاهش اثرات سوء افزايش مزمن هورمون كورتيزول در اثر سر و صدا را نشان مي‌دهد.
